# Genome-wide identification and functional analysis of the *ERF2* gene family in response to disease resistance against *Stemphylium lycopersici* in tomato

**DOI:** 10.1186/s12870-021-02848-3

**Published:** 2021-02-02

**Authors:** Huanhuan Yang, Yaoguang Sun, Hexuan Wang, Tingting Zhao, Xiangyang Xu, Jingbin Jiang, Jingfu Li

**Affiliations:** Laboratory of Genetic Breeding in Tomato, Key Laboratory of Biology and Genetic Improvement of Horticultural Crops (Northeast Region), Ministry of Agriculture and Rural Affairs, College of Horticulture and Landscape Architecture, Northeast Agricultural University, Harbin, 150030 China

**Keywords:** Tomato, Ethylene response factors, *ERF2*, Disease resistance, *S. Lycopersici*

## Abstract

**Background:**

APETALA2/ethylene responsive factor (AP2/ERF) transcription factors are a plant-specific family of transcription factors and one of the largest families of transcription factors. Ethylene response factors (ERF) regulate plant growth, development, and responses to biotic and abiotic stress. In a previous study, the *ERF2* gene was significantly upregulated in both resistant and susceptible tomato cultivars in response to *Stemphylium lycopersici*. The main purpose of this study was to systematically analyze the *ERF* family and to explore the mechanism of *ERF2* in tomato plants resisting pathogen infection by the Virus-induced Gene Silencing technique.

**Results:**

In this experiment, 134 *ERF* genes were explored and subjected to bioinformatic analysis and divided into twelve groups. The spatiotemporal expression characteristics of *ERF* transcription factor gene family in tomato were diverse. Combined with RNA-seq, we found that the expression of 18 *ERF* transcription factors increased after inoculation with *S. lycopersici*. In *ERF2*-silenced plants, the susceptible phenotype was observed after inoculation with *S. lycopersici*. The hypersensitive response and ROS production were decreased in the *ERF2*-silenced plants. Physiological analyses showed that the superoxide dismutase, peroxidase and catalase activities were lower in *ERF2*-silenced plants than in control plants*,* and the SA and JA contents were lower in *ERF2*-silenced plants than in control plants after inoculation with *S. lycopersici*. Furthermore, the results indicated that *ERF2* may directly or indirectly regulate *Pto*, *PR1b1* and *PR-P2* expression and enhance tomato resistance.

**Conclusions:**

In this study, we identified and analyzed members of the tomato *ERF* family by bioinformatics methods and classified, described and analyzed these genes. Subsequently, we used VIGS technology to significantly reduce the expression of *ERF2* in tomatoes. The results showed that *ERF2* had a positive effect on tomato resistance to *S. lycopersici*. Interestingly, *ERF2* played a key role in multiple SA, JA and ROS signaling pathways to confer resistance to invasion by *S. lycopersici.* In addition, *ERF2* may directly or indirectly regulate *Pto*, *PR1b1* and *PR-P2* expression and enhance tomato resistance to *S. lycopersici.* In summary, this study provides gene resources for breeding for disease resistance in tomato.

**Supplementary Information:**

The online version contains supplementary material available at 10.1186/s12870-021-02848-3.

## Background

Tomatoes are susceptible to various diseases that severely affect yield and quality. Gray leaf spot caused by *S. lycopersici* is one of the most devastating fungal diseases worldwide in tomato. However, plants have developed an elaborate signaling network to resist the invasion of pathogens by activating the expression of a series of resistance genes. In addition, transcription factors (TFs) play essential roles in regulating the expression of specific resistance-related genes in various defense response pathways. Ethylene responsive factors (ERF) belong to a subfamily of the AP2/ERF superfamily in plants. The *ERF* family is defined by the presence of a conserved *ERF* domain consisting of 58 or 59 amino acids containing an N-terminal, a three-stranded β-sheet, and a C-terminal α-helix. This family is widely involved in the regulation of plant development as well as in responses to abiotic and biotic stresses. To date, some members of the *ERF* family have been studied. Previous studies have identified *ERF* genes (*Pti4*/5/6 gene) that could bind to pathogenesis-related *Pto* protein kinases [1]. For instance, the overexpression of *Arabidopsis Pti4* could enhance resistance to *Pseudomonas* invasion by regulating the expression of GCC box-containing genes [2, 3]. At the same time, *Pti5* was isolated in previous studies of its physical interaction with the serine-threonine kinase encoded by the *Pto* gene [1]. Furthermore, a previous study indicated that overexpression of tomato *ERF2* could enhance basal resistance to *Pseudomonas syringae* pv. tomato [4].

APETALA2/ethylene responsive factor (AP2/ERF) transcription factors are a plant-specific family of transcription factors and one of the largest families of transcription factors. These transcription factors have a significant impact on plant growth and physiological activities and even affect evolution [5]. The three subfamilies AP2, ERF, and RAV constitute the AP2 superfamily, which contains more *ERF* family members than other subfamilies. The AP2 gene was first isolated in *Arabidopsis* and found to regulate flower development [6], and then the AP2 domain was detected in bacterial and viral HNH endonucleases [7]. Subsequently, *ERF* was discovered in tobacco and found to be present in four ethylene response binding proteins isolated from tobacco, namely, *ERF1*, 2, 3 and 4 [8]. *ERF* family proteins contain only one conserved domain, AP2, with 60 to 70 amino acid residues, and *ERF* can be divided into two subfamilies, *ERF* and *CBF*/*DREB*, according to differences in conserved amino acid residues and binding sequences. The DNA binding domain of the *ERF* subfamily specifically binds to the cis-acting element GCC-box with a conserved sequence of AGCCGCC [9, 10]. The ability of *ERF* to exert a positive or negative influence on the functional expression of downstream genes is based on the nucleotides in the GCC-box environment [11]. The DREBA subfamily can recognize the drought-induced element DRE (TACCGACAT) and the low-temperature-induced element CRT (AGCCGAC) [12], participate in the ethylene signaling pathway, and help plants resist the effects of stress [13, 14].

Salicylic acid (SA), ethylene (ET), and jasmonic acid (JA) have been identified as signaling molecules that play key roles in various defense response pathways. The SA pathway is antagonistic to the ET/JA pathway; however, *Pti4* and *AtERF1* are induced by SA as well as by the JA/ET pathway [15, 16]. Moreover, studies have shown that *Pti4*, *Pti5* and *Pti6* could indirectly regulate the SA response by interacting with other TFs in *Arabidopsis* [3]. *ERF* TFs were shown to regulate the expression of PR genes by binding to GCC (AGCCGCC) box-containing genes in their promoter regions [17]. Previously, *Pti4*/5/6 was shown to interact with the *Pto* gene and bind the GCC box to activate the expression of PR genes in the plant defense response to pathogens [1].

In this study, PlantTFDB was used to identify and analyze the 134 *ERF* transcription factor families in tomatoes, including their physical and chemical properties, evolutionary grouping, conserved motifs, gene structure, chromosome positions, protein tertiary structure, and tissue-specific expression. To further examine the role of the *ERF2* gene in the resistance to *S. lycopersici* in tomato, we used virus-induced gene silencing (VIGS) to downregulate *ERF2* gene expression in resistant tomato plants. In addition, we identified the potential signaling regulatory networks in which *ERF2* participates in resistance to *S. lycopersici*. In this study, we aimed to identify the role of *ERF2* in the response to *S. lycopersici* to provide a theoretical basis for cultivating resistant tomato varieties.

## Results

### Identification and analysis of the physical and chemical properties of *ERF* transcription factors

A total of 137 tomato *ERF* genes were confirmed with the SMART (http://smart.emblheidelberg.de/smart/batch.pl) and CDD (https://www.ncbi.nlm.nih.gov/Structure/bwrpsb/bwrpsb.cgi) online tools. Genes without complete AP2/ERF domains were discarded. Finally, 134 transcription factors were screened. As shown in Supplementary Table 1, the longest sequence and the heaviest molecular weight were observed for *Solyc04g071770.2.1*, at 452 aa and 49,582.81, respectively; the shortest sequence and the lightest molecular weight were observed for *Solyc10g080310.1.1*, at 73 aa and 8382.60, respectively. The isoelectric point ranged from 4.09 (*Solyc10g076380.1.1*) to 10.08 (*Solyc10g080310.1.1*). The instability coefficient ranged from 21.51 (*Solyc12g038450.1.1*) to 86.25 (*Solyc01g090340.2.1*). Among the transcription factors, members *Solyc03g093530.1.1*, *Solyc06g050520.1.1*, *Solyc06g063070.2.1*, *Solyc10g080310.1.1*, *Solyc10g080650.1.1*, *Solyc12g038440.1.1*, and *Solyc12g038450.1.1* all had instability coefficients below 40, indicating that they were more stable than the others. The total average hydrophilicity ranged from − 1.122 (*Solyc06g068830.1.1*) to − 0.303 (*Solyc03g006320.1.1*), indicating hydrophilic proteins.

### Phylogenetic tree of *ERF* transcription factors

**Fig. 1:**
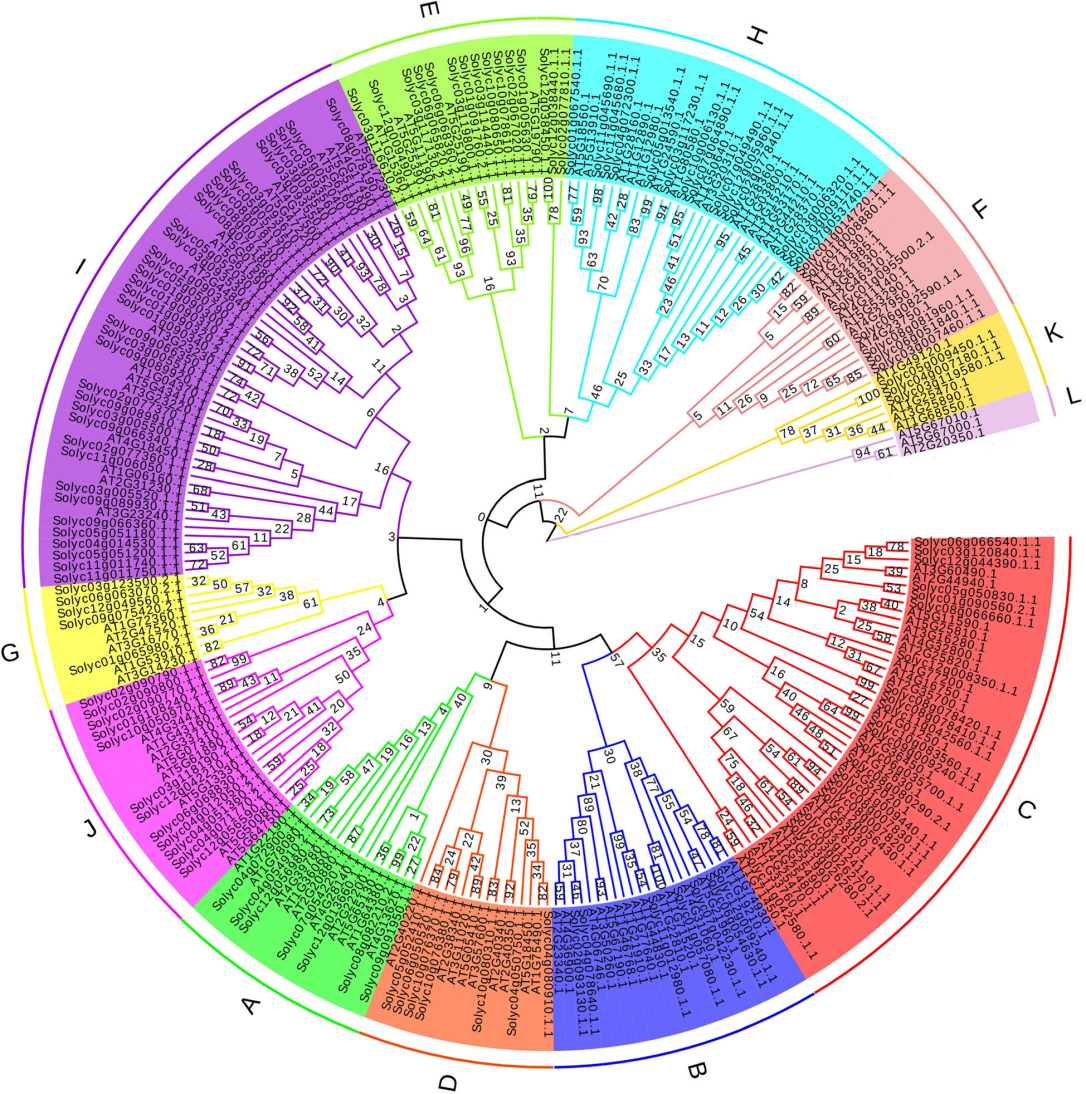
Phylogenetic tree of *ERF* proteins from tomato and *Arabidopsis thaliana*. Each background color and the letters of the outer ring represent a different branch

A total of 134 tomato *ERF* transcription factors and 122 *Arabidopsis ERF* genes were combined to construct a comprehensive phylogenetic tree. According to the grouping by conserved domain and the grouping of *ERF* family genes in *Arabidopsis*, the tomato and *Arabidopsis ERF* transcription factor genes in this experiment were divided into 12 groups (Fig. 1). Among these groups, groups A, B, C and D contained members of the *CBF*/*DRBE ERF* subfamily, corresponding to A6, A5, A1/A4 and A2 in the *Arabidopsis* group, respectively. There were no A3-subfamily genes in the *ERF* family of tomato, proving that there were no genes with a structure and function similar to those of *AT2g40220.1* in tomato. In previous studies, 12 *ERF* genes were classified into group B6 in *Arabidopsis thaliana* [18, 19]. Based on motif analysis, B6 genes were divided into three groups. Therefore, in this experiment, B6 was divided into three groups (E, K, and L) as suggested by previous research, and there were no tomato *ERF* genes in group L. Groups F, G, H, I, and J corresponded to the B5, B2, B1, B3, and B4 groups in *Arabidopsis*, respectively. A total of 43 genes belonged to the CBF/DRBE subfamily, and 92 genes belonged to the *ERF* subfamily. Group I contained the most tomato *ERF* genes, with 35.

### Conserved motif analysis and gene structural analysis of *ERF* transcription factors

**Fig. 2:**
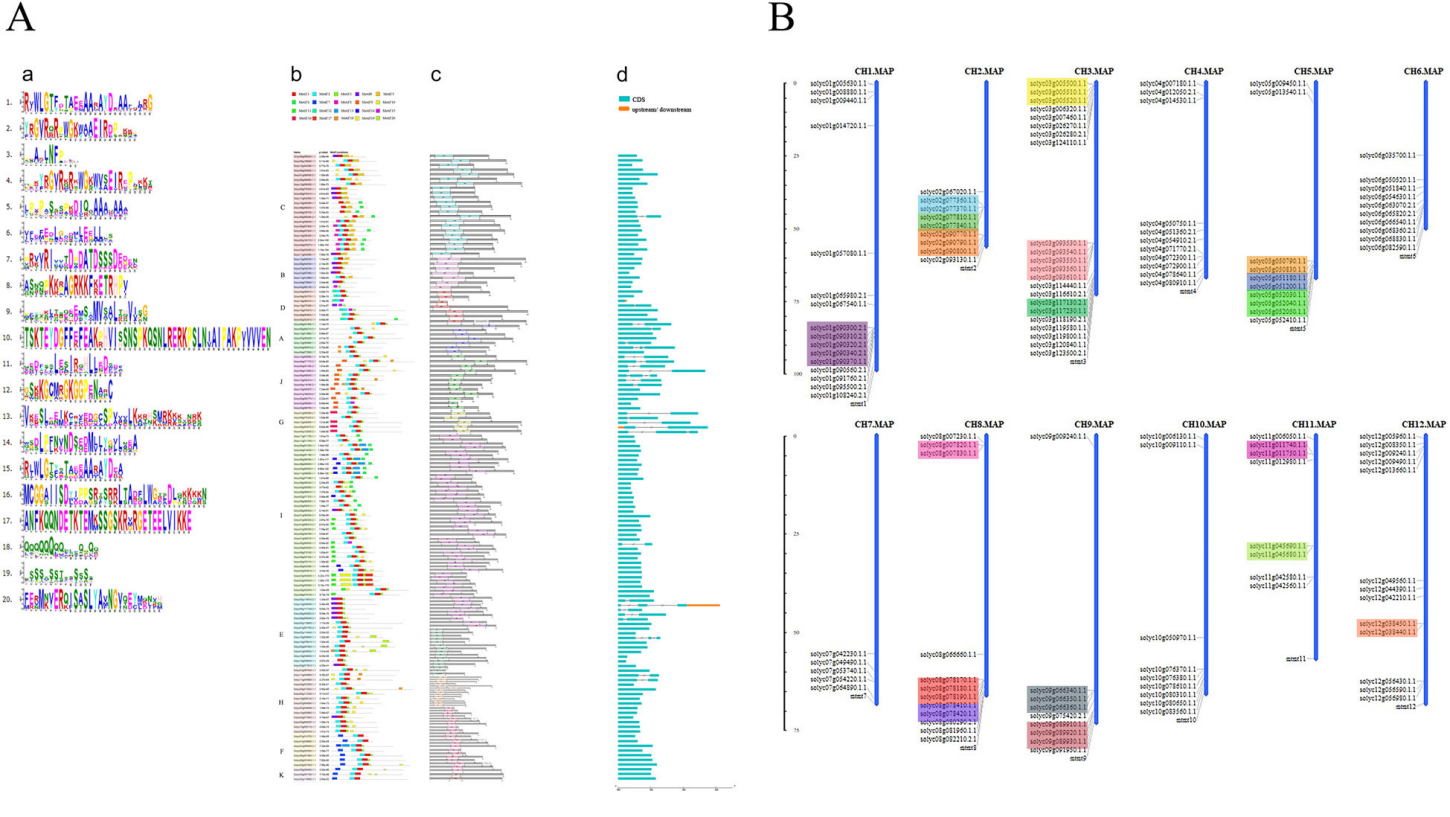
The structure and chromosomal locations of *ERF* genes in tomato. **A** The structure of *ERF* genes in tomato. (a) Motif logo; (b) Distribution of conserved motifs on each *ERF* genes in tomato; (c) The position of the AP2 conserved domain on the *ERF* genes; (d) Distribution of exons and introns in the *ERF* genes. **B** Chromosomal locations of the tomato *ERF* genes. The scale was used to estimate the length of chromosomes, and the same set of tandem replication genes was marked with the same background color

To understand the specific distribution of conserved motifs of tomato *ERF* genes, 20 conserved motifs were identified by the online MEME analysis tool (http://meme-suite.org/). The logos of the 20 conserved motifs found are shown in the figure, and their position information in each subgroup is shown in Fig. 2A-a. On average, each member contained 4 motifs, and *Solyc11g006050.1.1* had the largest number of motifs, which was 7 (Fig. 2A-b). The results showed that the conserved AP2 domains constituted by motifs 1, 2 and 3 were the most conserved in the sequences of tomato *ERF* transcription factors, which together with other conserved elements contributed to the diversity and identity of the genes. Among these transcription factors, 18 members of the C, B, D, E and H subgroups lacked motif 2. Similarly, most genes of subgroups B, D, J and I contain motif 15. Motif 5 was detected in groups C and B. Motif 6 appeared in members of subgroups C, D, and I and was conserved at the C-terminus of the protein sequence. In both groups F and K, motif 7 was identified as conserved at the N-terminus of the sequence. Motifs 8/9/12/16/20 were unique to the C/J/D/G/E group. Group I had the most members and, correspondingly, the most characteristic motif, motif 10/11/13/14/17. The results show that the members of the same subgroup are similar in rank and position, and the unique conserved motifs in different subgroups also enhance the support of the phylogenetic tree. The distribution of the conserved structural domain of the tomato *ERF* family is shown in Fig. 2A-c. Most of the *ERF* members have only AP2, a conserved structural domain with a length of approximately 60–70 amino acids. For example, *Solyc05g052410.1.1* and *Solyc08g081960.1.1*, in addition to having AP2, also contain the H^+^-ATPase subunit H (NtpH) superfamily and Flavodoxin domain, respectively. The name of the *Solyc02g077810.1.1* conserved domain is the same as that in the AP2 superfamily. The more closely related the members of an evolutionary branch are, the more closely related their conserved domains are, and the more similar their biological functions are. The dissimilarity in the arrangement of conserved domains among the members of the same subgroup may be caused by evolutionary or recombination mutations in the progeny.

To further study the gene structure of the *ERF* transcription factor family, a structural distribution map of introns and exons of 134 members was obtained through the Gene Structure Display Server (GSDS) analysis platform (http://gsds.cbi.pku.edu.cn/) (Fig. 2A-d). As shown in the figure, most members of the tomato *ERF* family contain only exons (107/134, 79.8%). This structural feature is similar to that in the *Arabidopsis ERF* family. In addition, no intron was found in the B, F, and K subgroups, and only one intron was found in the C, D, and I subgroups. The smaller number of intron-containing members than of non-intron-containing members in the tomato *ERF* family may be due to an increase in or a loss of introns during evolution.

### Distribution of tomato *ERF* transcription factors on chromosomes

The 134 *ERF* transcription factors in tomato showed an uneven distribution on 12 chromosomes (Fig. 2B). Chromosome 3 contained the most members, with 22 members. Chromosome 7 contained the fewest, with only five members. A total of 16, 9, 22, 11, 10, 10, 5, 12, 9, 9, 8, and 13 *ERF* genes were distributed sequentially on chromosomes 1–12 in tomato. A tandem repeat was defined as adjacent genes on the same chromosome within 100 kb. There were 20 pairs of genes in the tomato *ERF* family that exhibited tandem replication, and the number of tandemly replicated genes on chromosome 3 was the highest, at 4 pairs, including a total of 13 genes. Tandem replication led to the production of multiple gene clusters. The members of the *ERF* family accounted for 39.5% of the total, and 75% of the chromosomes of the tomato family exhibited tandem duplication.

### Expression of the tomato *ERF* transcription factor family in different organs

**Fig. 3:**
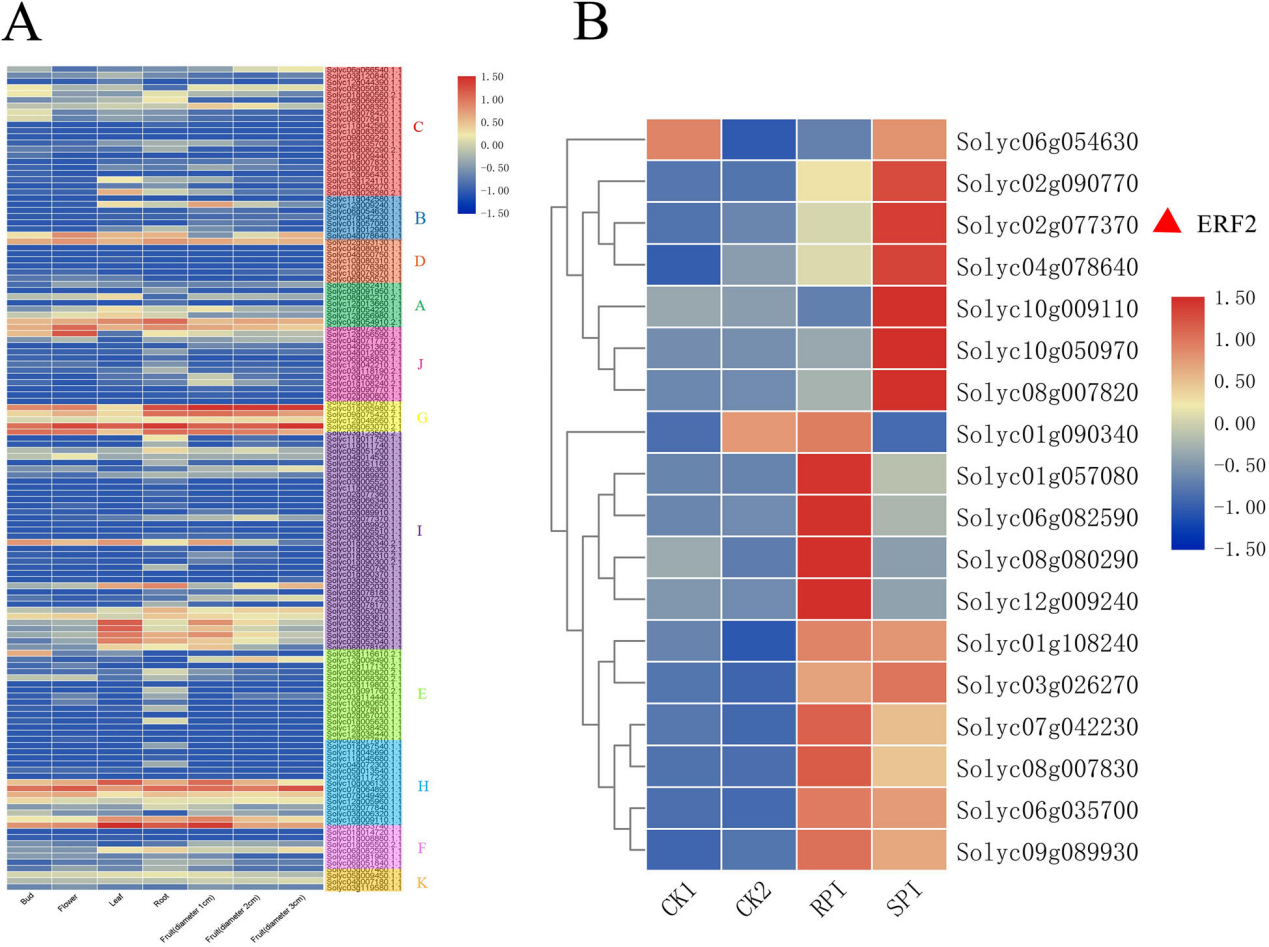
Expression pattern map of *ERF* genes in tomato. **A** Expression pattern of the *ERF* gene in different organs of tomato. **B** Comparison of differential expression of 18 *ERF* genes in tomato inoculated with *S. lycopersici*. Red triangles represent *ERF2* gene. The colors from blue to red represent the range of the relative expression levels from low to high

To better understand the role of *ERF* genes in tomato development, we used previously published tomato RNA-seq data to draw a heat map of *ERF* tissue-specific expression (Fig. 3a). The results showed that the expression of most of the genes in the tomato *ERF* family was low in the bud, flower, leaf, root and fruit of tomato. The expression levels of *Solyc06g063070.2.1*, *Solyc03g123500.2.1*, and *Solyc07g064890.1.1* in tomato seedlings were similar and higher than those of other genes. *Solyc06g063070.2.1* had the highest expression level in flowers. The *Solyc12g056590.1.1*, *Solyc07g064890.1.1*, *Solyc04g072900.1.1*, and *Solyc03g123500.2.1* genes were highly expressed in flowers. *Solyc07g053740.1.1* had the highest expression in leaves. The *Solyc10g006130.1.1*, *Solyc03g093540.1.1*, *Solyc03g093550.1.1*, *Solyc06g063070.2.1*, *Solyc03g093560.1.1*, and *Solyc05g052040.1.1* genes also had higher expression levels in leaves. *Solyc06g063070.2.1* was also the most highly expressed gene in the root. The *Solyc01g065980.2.1*, *Solyc07g053740.1.1*, *Solyc07g064890.1.1*, *Solyc04g054910.2.1*, and *Solyc09g075420.2.1* genes showed higher expression in the roots. During the fruit expansion period, the expression of some genes decreased with increasing fruit diameter, while that of others showed the opposite trend. The *Solyc01g065980.2.1* gene had the highest expression level in fruits with a diameter of 1 cm. As the fruit gradually matured, the expression level of this gene decreased. The expression of the *Solyc06g063070.2.1* gene showed an upward trend as the fruit matured, with the highest expression in the fruits with a diameter of 3 cm.

### Expression pattern of *ERF* transcription factors in tomato inoculation with *S. lycopersici*

In this study, we screened 18 *ERF* genes based on transcriptome data. These genes were grouped into two groups. The results showed that except for *Solyc06g054630* and *Solyc01g090340*, the expression of 16 *ERF* genes retrieved in the RNA-seq data increased after inoculation (Fig. 3b). It is worth noting that in the first group, the difference was mainly shown in CK2 and SPI, while in the other group, it was shown in CK1 and RPI. This shows that these *ERF* genes play a positive role in tomato resistance to pathogen infection.

### Phylogenetic analysis and sequence alignment of *ERF2*

**Fig. 4:**
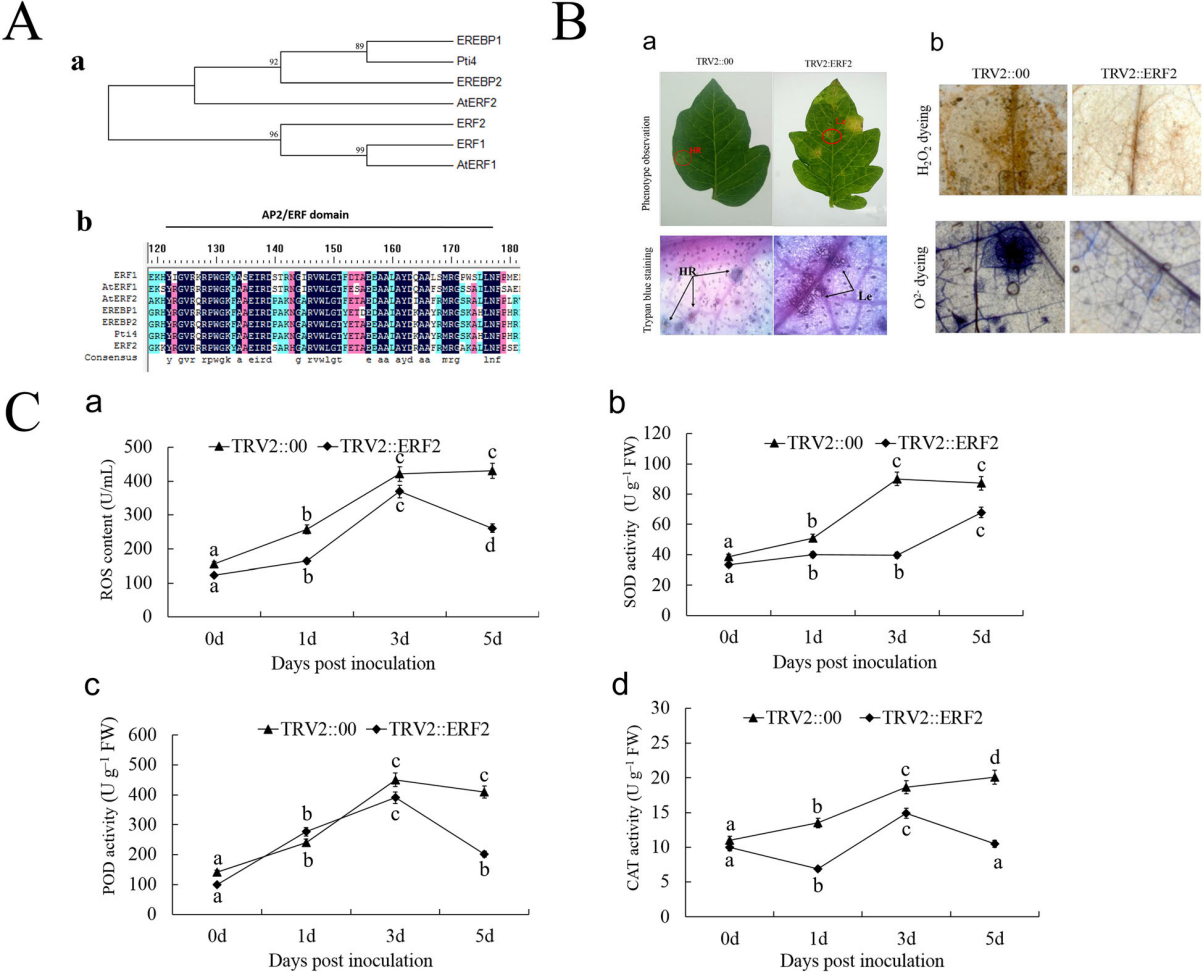
Sequence alignment, phenotype, and enzyme activity analysis of *ERF2* in tomato. **A** Phylogenetic tree and sequence alignment of *ERF2*. (a) Phylogenetic tree of *ERF2* and other *ERF* proteins; the phylogenetic tree was constructed via amino acid sequences of the AP2/ERF domain. (b) Alignment of *ERF2* with other *ERF* proteins. *ERF2* is composed of an ERF domain. The black and light-gray colors represent identical and conserved amino acids, respectively, and the darker blue colors represent greater percentages of the same amino acid. **B** Phenotypic and physiological changes after silencing of the *ERF2* gene. (a) Silencing of *ERF2* decreased disease resistance in tomato plants. The *ERF2-*silenced plants exhibited disease symptoms with lesions on leaves at 3 dpi, and only a hypersensitive reaction without disease symptoms was observed in TRV2 empty vector plants. (b) Histopathological observation of the accumulation of H_2_O_2_ and O^2−^. HR, hypersensitive reaction; Le, lesions. C, ROS content (a) and SOD (b), POD (c) and CAT (d) activities in tomato plants after inoculation with *S. lycopersici* at different time points. The data presented in (**C**) are the means ± SD from three independent experiments, and different letters above the columns indicate significant differences at the *p* < 0.05 level

The coding sequence of *ERF2* has one AP2/ERF domain, and this protein belongs to the *ERF* TF B-3 family (Fig. 4A-a). In addition, *ERF2* is closely related to tomato *ERF1* and *A. thaliana AtERF1*. The results indicated that *ERF2* may have a similar function to other B-3 family members in plants. Analysis of the conserved protein sequence database revealed that *ERF2* shares high similarity with other *ERF* proteins in terms of their whole putative protein sequences (Fig. 4A-b).

### *ERF2*- silenced plants showed impaired disease resistance to *S. lycopersici*

To investigate whether *ERF2* influences tomato plant defense against *S. lycopersici*, we performed VIGS to downregulate *ERF2* gene expression. In order to prevent interference with the expression of other *ERF* genes, the target fragment we selected is shown in Supplementary Figure 1. The results showed that disease symptoms were observed in the *ERF2*-silenced plants compared to the TRV2 empty vector plants after inoculation with *S. lycopersici*. In the *ERF2*-silenced plants, the lesions were aggravated, and perforations were observed. In contrast, only a hypersensitive reaction (HR) without disease symptoms was observed in the TRV2 empty vector plants (Fig. 4B-a). These results indicated that silencing the *ERF2* gene in resistant tomato plants could impair resistance to *S. lycopersici.*

As shown in Fig. 4B-a, low levels of mycelial hyphae and a weak HR with necrotic lesions were observed in the *ERF2*-silenced plants. Nevertheless, strong HR symptoms without hyphal growth were observed in the TRV2::00 empty vector plants. Therefore, these results indicated that the HR was impaired in the *ERF2*-silenced plants compared to the TRV2::00 empty vector plants at 3 dpi with *S. lycopersici*.

### Accumulation of H_2_O_2_ and O^2−^ was impaired in the *ERF2*-silenced plants

The accumulation of H_2_O_2_ and O^2−^ can be used to evaluate the effects of disease resistance in tomato plants. At 3 dpi, H_2_O_2_ accumulation was too weak to observe in the TRV::*ERF2* plants compared to the TRV::00 empty vector plants. H_2_O_2_ was observed earlier and was more abundant in the TRV::00 plants than in the TRV::*ERF2* plants (Fig. 4B-b). Based on these results, we concluded that the downregulation of *ERF2* gene expression could decrease resistance to *S. lycopersici* in tomato plants.

### ROS content and SOD, POD and CAT activity assays

ROS production and enzyme activities were detected over a time course; therefore, leaves at 0, 1, 3 and 5 dpi were collected for determination of the ROS content and the SOD, POD and CAT activities. Inoculation with *S. lycopersici* caused the ROS content and SOD, POD and CAT activities to sharply increase at 3 dpi (Fig. 4C). In particular, in the *ERF2*-silenced plants, the ROS content and SOD, POD and CAT activities were lower than those in the control plants at 1, 3 and 5 dpi.

### *ERF2* may enhance disease resistance to *S. lycopersici* through SA and JA signaling pathways

**Fig. 5:**
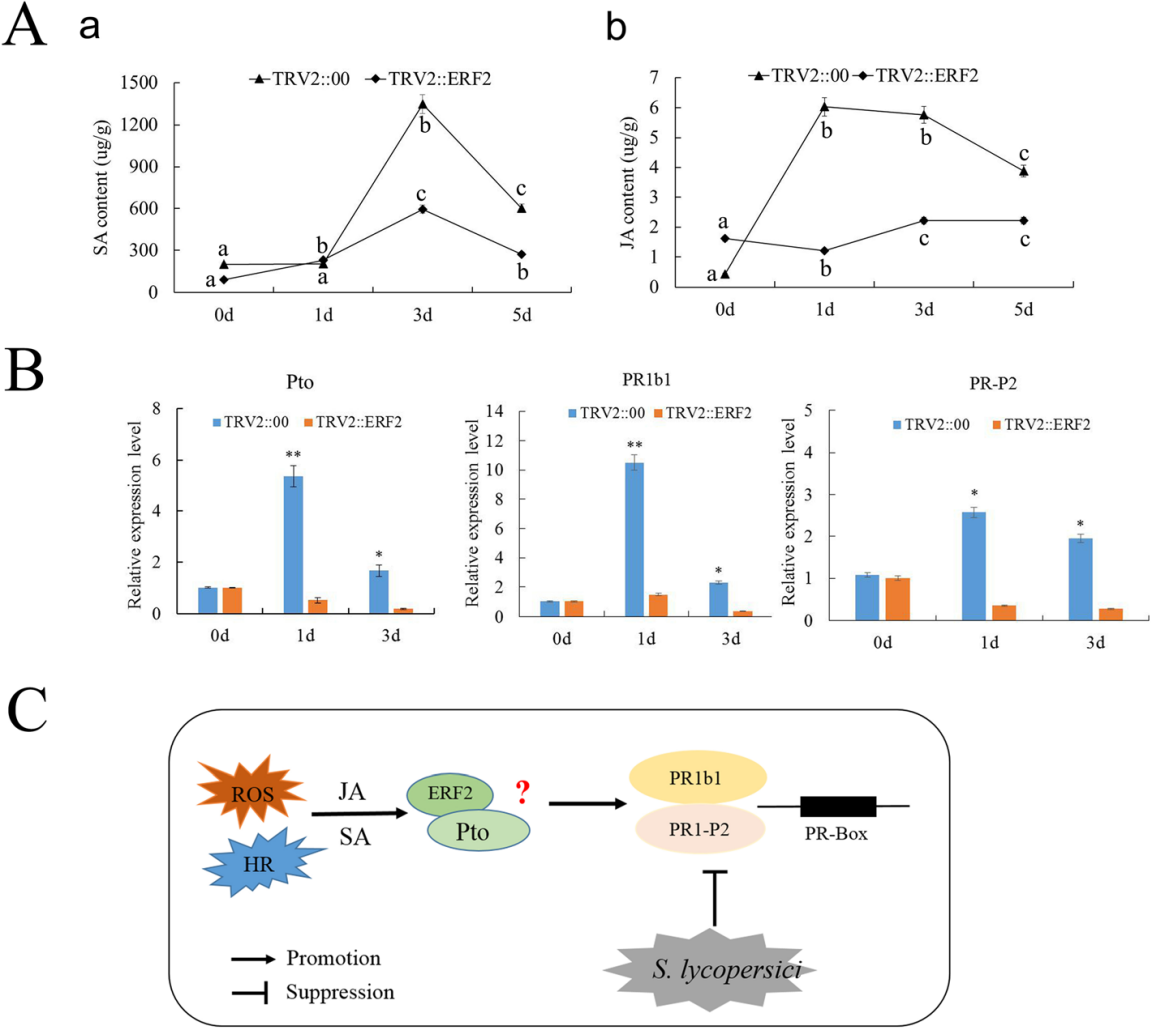
Expression of SA and JA, expression of related genes and predictive expression model. **A** SA (a) and JA (b) hormone levels in the *ERF2-*silenced plants. The data presented in (**A**) are the means ± SD from three independent experiments, and different letters above the columns indicate significant differences at the *p* < 0.05 level. **B** Silencing of *ERF2* decreased the expression levels of the *Pto* and *PR* genes after infection with *S. lycopersici*. TRV::00, empty vector plants; TRV::*ERF2*, *ERF2-*silenced plants. The asterisks indicate significant differences in the expression levels between the silenced lines and the control lines (**p < 0.01; *p < 0.05, Student’s t-test). **C** Hypothetical model for the *ERF2*-mediated defense response to *S. lycopersici*

To analyze the hormonal response to *S. lycopersici* infection, we performed liquid chromatography-mass spectrometry (LC-MS) to measure the JA and SA contents in the *ERF2*-silenced and TRV::00 plants. For SA, the content in TRV2::*ERF2* and TRV::00 plants peaked at 3 days, and the content of the latter was 4.7 times greater than that of the former. The JA levels of the *ERF2*-silenced plants were significantly lower than those of the TRV::00 plants after inoculation with *S. lycopersici* (Fig. 5A). These results indicated that *ERF2* probably participates in both the SA and JA signaling pathways to improve disease resistance to *S. lycopersici* in tomato plants.

### *ERF2*-silencing decreased the *Pto* and *PR* gene expression levels

Previous studies have shown that Pti4/5/6 interacts with *Pto* to regulate disease resistance. In addition, many studies have shown that *ERF* genes regulate the expression of PR genes to enhance plant resistance to disease [20, 21]. Here, qRT-PCR was used to identify the regulatory relationship between *ERF2* and the defense genes *Pto* and *PRs*. As shown in Fig. 5B, the expression levels of the *Pto, PR1b1* and *PR1-P2* genes were significantly decreased in the *ERF2*-silenced plants compared to the TRV::00 plants after inoculation with *S. lycopersici.* Therefore, we proposed that *ERF2* enhances disease resistance to *S. lycopersici* by directly or indirectly regulating the expression of the *Pto* and PR genes in tomato plants.

In particular, studies have indicated that the HR and the accumulation of ROS are stronger in resistant cultivars than in susceptible cultivars, leading to improved disease resistance [22]. Consistent with these previous studies, our studies showed that downregulating the gene expression of *ERF2* decreased HR-induced cell death, the production of H_2_O_2_, and O^2−^ in the *ERF2*-silenced plants compared to the TRV::00 plants. These results indicated that the accumulation of ROS was positively correlated with the HR in the disease resistance to *S. lycopersici.*

Many studies have shown that the regulation of PR gene expression by *ERF* TFs requires the combination of GCC-box or DRE/CRT cis-acting elements [23, 24]. In addition, studies have shown that different sequences on the GCC-box side affect the binding efficiency of *ERFs*, indicating that various *ERFs* may regulate different gene sets [25]. *PR-P2* and *PR1b* are representative marker genes of the JA/ET- and SA-mediated defense signaling pathways. In particular, the tomato *Pto* gene could enhance defense responses after inoculation with *P. syringae* pv. *tabaci* [26]. The overexpression of the tomato *Pto* gene could activate the expression of PR gene resistance to *Pseudomonas* species, and EREBPs interacted with the *Pto* protein to regulate disease resistance [20]. Here, our studies showed that downregulation of *ERF2* gene expression could decrease the *Pto*-mediated resistance to *S. lycopersici.* Furthermore, *Pti4*/5/6 TFs bind to the PR box to regulate gene expression. Similarly, our studies also showed that silencing the *ERF2* gene decreased the gene expression of *PR1b1* and *PR-P2*. Together, these results indicate that *ERF2* may directly or indirectly regulate *Pto*, *PR1b1* and *PR-P2* expression and enhance tomato resistance to *S. lycopersici*. However, it remains to be determined whether *ERF2* interacts with the *Pto* protein to regulate PR gene expression and enhance the resistance of tomato to *S. lycopersici* (Fig. 5C).

Furthermore, previous studies have also shown that SA and JA are important signaling molecules involved in PTI and ETI, regulating plant diseases and responses to abiotic stresses [27, 28]. In addition, the SA and JA/ET signaling pathways can induce defense responses, including the expression of most *PR* proteins [29–31]. Our data were consistent with previous findings that the SA and JA contents were decreased in *ERF2*-silenced plants compared to TRV::00 plants, suggesting that *ERF2* involvement in the resistance of tomato plants to *S. lycopersici* may be dependent on the SA and JA signaling pathways.

## Discussion

Ethylene is one of the most important hormones in plants, and its physiological functions affect plants through a series of physiological activities during their growth and development. Ethylene receptors regulate the downstream *ERF* and stimulate the expression of related genes through signal mediation [32]. The *ERF* family, a large family of transcription factors, is unique to plants. To date, *ERF* transcription factors have been identified in a variety of plant fruits. Its family members have conserved characteristics in each plant. Phylogenetic grouping also reveals similarities, but the number of genes varies: *Arabidopsis* has 122 genes [19], rice has 131 [18], corn has 133 [33], wheat has 104, apple has 51 [34], and *Brassica napus* has 286 [35]. In some species, individual subgroups do not exist, no lower plants contain singletons, and some higher plants contain singletons. All the groups studied belong to the dicotyledon or monocotyledon plant system, so it is speculated that the *ERF* transcription factor family completed differentiation before the split of dicotyledons and monocotyledons. Gene mutation, chromosome exchange and gene loss during the evolution of species are all reasons for the expression differences and functional diversity of the *ERF* family in different species.

In this experiment, 137 tomato *ERF* genes were obtained using a plant transcription factor database. In the detection of conserved domains, 134 genes met the requirements, so they were excavated and subjected to bioinformatic analysis. This experiment divided 134 *ERF* members into twelve groups based on the conserved domain of the genes. Sakuma divided *Arabidopsis ERF* members into A1-A6 (belonging to the DREB subfamily) and B1-B6 (belonging to the *ERF* subfamily). Nakano divided *Arabidopsis ERF* family members into two groups based on those of Sakuma, namely, the ten groups I-X and the class VI (VI-L) and class Xb (Xb-L) groups. This experiment adopted a grouping method similar to that of Nakamo. Group VI-L corresponds to group K, and the Xb-L group does not contain homologous tomato *ERF* genes. Genes in the same clade have the closest kinship and likely perform similar or complementary physiological functions. A preliminary understanding of the function of tomato *ERF* genes in the same clade or the same group can be obtained by understanding the function of the corresponding *Arabidopsis ERF* genes. The conserved domains involved in the regulation of plant growth and development in transcription factors always consist of different conserved motifs. In this study, motifs 1, 2, 3, 4, and 15 corresponded to the conserved part of the AP2 domain, and the most conserved elements were WLG and YRG. In addition to the conserved domain of AP2, transcription factors in the same subgroup also included one or more specific motifs, which may be related to different regulatory functions of *ERF* members, reflect the functional diversity of the *ERF* family, and have the potential to promote the interaction between nuclear localization and proteins [36]. The *ERF* family is largely free of introns, as has been demonstrated in several species [18, 35]. Taking *Arabidopsis* as an example, only a few more than 20 genes contain introns, which is similar to the conclusion of this study, and the introns are located in a conserved position, which verifies the reliability of grouping. The lack of introns may be due to the absence of intron transposons or intron loss during evolution. When plants complete evolution, gene replication often occurs, which expands and enriches the number and function of genes in the genome. The results of this study showed that 53 genes on 9 chromosomes exhibited tandem replication. This shows that the expansion of the *ERF* family mainly depends on tandem repeats. The homology modeling results of the tertiary structure of *ERF* proteins show that the tertiary structures of the proteins in the same subfamily are similar, and those of the genes in different subfamilies are different due to variable spatial angles. However, overall, the spatial structure of the AP2 domain is composed of three antiparallel β-sheets parallel to the β-sheet α-helix, which is the same as a previous conclusion [37].

Previous studies have shown that AP2/ERF proteins play an important role in the transcriptional regulation of various biotic stress responses. In addition, B-subfamily genes have been shown to be involved in resistance to various diseases [18], and B-3 subfamily members were reported to regulate plant disease resistance [38]. In this study, phylogenetic analysis showed that *ERF2* belonged to the B-3 subfamily of the *ERF* protein family, and *ERF2* showed a close relationship to *ERF1* and *AtERF1*. Previous studies have demonstrated that *ERF1* and *AtERF1* play a role in disease resistance responses. In this study, our results showed that downregulating the gene expression of *ERF2* impaired disease resistance to *S. lycopersici,* and obvious disease lesions were observed on the *ERF2*-silenced plants compared with the TRV::00 plants.

## Conclusions

In this study, we identified and analyzed the members of the tomato *ERF* family by bioinformatics methods and then classified, described and analyzed these genes. A total of 134 *ERF* genes were divided into 12 branches, and genes in the same branch had similar gene structure. The expression of these genes in different organs of the tomato plant was specific. We found that *ERF2* was an AP2/ERF TF that positively regulated tomato plant resistance to *S. lycopersici* by VIGS. Interestingly, *ERF2* played a key role in multiple SA, JA and ROS signaling pathways to confer resistance to invasion by *S. lycopersici.* In addition, *ERF2* may directly or indirectly regulate *Pto*, *PR1b1* and *PR-P2* expression and enhance tomato resistance to *S. lycopersici.* In summary, this study provides gene resources for breeding for disease resistance in tomato plants.

## Methods

### Plant materials

Tomato-resistant cultivars (cv. Motelle) were provided by the Chinese Academy of Agricultural Sciences. All tomato plants were grown in an artificial climate chamber with a light-dark (LD) cycle (16 h L: 8 h D), and the light condition was as follows: light intensity 40,000 Lx, temperature 24 °C, and relative humidity 60%; the dark condition was as follows: temperature 16 °C and relative humidity 50%. *S. lycopersici* was plated on potato dextrose agar (PDA) at approximately 28 °C for 2 weeks until spores were produced.

### Identification of tomato *ERF* transcription factor family members

The protein sequences of tomato *ERF* transcription factor family genes were downloaded from PlantTFDB (http://planttfdb.cbi.pku.edu.cn). According to Pfam PF00847 of the tomato *ERF* transcription factor AP2 domain obtained from the Pfam database, all sequences were identified by using the online protein structure prediction tool SMART (http://smart.embl-heidelberg.de/), and genes without the AP2 domain were deleted. On the ExPASy website, the physical and chemical properties, such as protein length and molecular mass, of the protein amino acid sequences of all tomato *ERF* transcription factors screened were predicted.

### Phylogenetic analysis of the tomato *ERF* transcription factor family

We introduced the sequences of the *Arabidopsis ERF* transcription factor family as a reference for tomato *ERF* grouping when constructing a phylogenetic tree of the tomato *ERF* transcription factor family. The 139 amino acid sequences of the *Arabidopsis ERF* transcription factor family were obtained from the PlantTFDB database. Duplicate genes and genes without conserved domains were excluded. Multisequence alignment of the conserved AP2 domains in the *ERF* family of tomato and *Arabidopsis thaliana* was performed using ClustalW, and the results were imported into MEGA 7.0 software to construct a rootless evolutionary tree of the *ERF* family. The algorithm used was the neighbor-joining (NJ) model, the bootstrap value for verification was set to 1000, and the model selection parameter was p-distance. The evolutionary tree was edited online with EvolView v3 (https://www.evolgenius.info/evolview) [39].

### Structural analysis of the tomato *ERF* transcription factor family

Conserved motif analysis of the amino acid sequences of the tomato *ERF* family was performed online via MEME (http://meme-suite.org/). The maximum number of search motifs was set to 20, and the amino acid width was set to 6–50. The basic information on the conserved domain of the tomato *ERF* family was obtained from the Conserved Domains Database (CDD) of the NCBI, and the conserved domain was mapped by DOG 2.0 software (http://dog.biocuckoo.org/index.php) [40]. The GSDS (http://gsds.cbi.pku.edu.cn/) online analytical function was used to obtain the tomato *ERF* transcription factor family exon and intron genetic structure patterns. Coding sequence (CDS) and genome sequence information for the tomato *ERF* transcription factor family was obtained from SGN (https://solgenomics.net/).

### Chromosome locations of the tomato *ERF* transcription factor family

After obtaining the location information of the *ERF* family on twelve tomato chromosomes from the SGN database, MapInspect software was used to complete the tomato *ERF* family chromosome location map (http://www.plantbreeding.wur.nl/UK/software_mapinspect.html). Tomato *ERF* genes with serial replication were identified.

### Protein tertiary structural analysis of the tomato *ERF* transcription factor family

Through the online analysis software SWISS-MODEL (https://swissmodel.expasy.org/) [41], all genes of the tomato *ERF* transcription factor family were homologously modeled to analyze and predict the tertiary structure of *ERF* family proteins.

### Analysis of the expression patterns of the tomato *ERF* transcription factor family

We obtained tomato Illumina RNA-seq data from SGN and NCBI (SRP097450). Fragments per kilobase of exon model per million mapped reads (FPKM) values were used to represent the expression levels of *ERF* genes. We selected the transcriptome data of genes belonging to the *ERF* transcription factor family. Using the Heinz variety as an example, using TBtools software (https://github.com/CJChen/TBtools) [42], log FPKM values with log10 as the base were calculated, a heat map was drawn, and then the *ERF* genes in tomato tissue were analyzed. Expression levels were also analyzed. The specific tissues included buds, flowers, leaves, roots, and fruits. Regarding the infection of pathogens, the expression profile of the *ERF* gene conformed to the standard of high probability value (*p* > 0.8).

### VIGS vector construction and agroinfiltration

The specific primers designed by the SGN VIGS Tool were amplified to prevent interference with the expression of other *ERF* genes (https://vigs.solgenomics.net/). The PCR protocol was as follows: 94 °C for 10 min; 40 cycles of 5 s at 94 °C, 15 s at 65 °C, and 30 s/kb at 72 °C; and 72 °C for 10 min. The amplified 300 bp PCR product and TRV2 empty vector were digested with the restriction enzymes EcoRI and BamHI. Then, the target fragments were ligated into the TRV2 empty vector. The constructs were transformed into competent *Escherichia coli* DH5α, and single clones were cultured in liquid LB containing 50 μg/mL kanamycin. Once the recombinant plasmids were confirmed by sequencing, they were transformed into *Agrobacterium tumefaciens* strain GV3101 and shaken to optical density = 0.25 at 28 °C and 200 rpm. In addition, TRV-*PDS* (phytoene desaturase) was used as a control for evaluation of VIGS [43]. *A. tumefaciens* cells containing TRV1 were mixed with those containing TRV2-derived constructs or TRV2 empty vector at a volume ratio of 1:1. Briefly, 14-day-old Motelle plants were vacuum infiltrated with TRV-*PDS*, TRV-*ERF2* and TRV-00 syringes containing approximately 0.5–1 mL of *Agrobacterium* cells and kept at 22 °C in a growth chamber with a 12-h photoperiod.

### Pathogen inoculation and phenotypic observation

Tomato plants were inoculated with TRV-*PDS*, TRV-*ERF2* and TRV-00 at the age of 4 weeks. The treatment group plants were inoculated with 250 mL conidia suspension (1 × 10^4^ spores/mL), and the control group plants were sprayed with the same amount of sterilized water. Each of these groups contained 10 plants with the same growth potential. The plants were kept in a light culture chamber (light: 16 h, 28 °C; dark: 8 h, 25 °C) with a relative humidity of 80%. The disease status of plants was observed continuously after inoculation, and the leaves were collected at 0 and 3 days post inoculation (dpi). Furthermore, the photobleaching phenotype of the PDS gene acted as the positive control for evaluation of VIGS. The TRV2:00 empty vectors were included as controls.

### Microscopic observation

At 0 and 3 dpi, 0.1% trypan blue (TB) staining was used to confirm the disease status of tomato plants infected with *S. lycopersici* [44]. Similarly, 0.1% 3,3′-diaminobenzidine (DAB) and 0.2% nitrotetrazolium blue chloride (NBT) were used to detect the accumulation of H_2_O_2_ and O^2−^ in plant leaves, respectively [45, 46]. Finally, an optical microscope was used to record these images.

### qRT-PCR analysis and physiological index determination

qRT-PCR was performed with three independent biological replicates using AceQ® qPCR SYBR® Green Master Mix (Vazyme, Nanjing, China) in a 20 μL volume on a qTOWER3G Real-time System (Analytik Jena AG, Germany). The qRT-PCR primers are listed in Supplementary Table 2. *EF1α* was used as an internal control for normalization of the data. Relative expression was calculated using the 2^–△△^CT method.

The activities of the main disease-resistance enzymes, including ROS, SOD, POD, and CAT, were determined using the ROS Assay Kit E004–1-1, SOD Assay Kit A001–3-2, POD Assay Kit A084–3 and CAT Assay Kit A007–1-1 (Nanjing Jiancheng Bioengineering Institute, Nanjing, China) with the protocols provided by the manufacturer. The leaves we collected are random and they’re mixed together to extract total RNA. The leaves were collected at 10:00 AM on the 0, 1, 3 and 5 day after inoculation, and the collected samples were immediately used for analysis. All the treatment groups were carried out at the same time, and the whole experiment was repeated three times.

### Assay of SA and JA contents

High-performance liquid chromatography (HPLC) was used to determine the levels of the endogenous hormones SA and JA [47]. The leaves of the plants were collected at 10:00 AM the 0, 1, 3 and 5 day after inoculation, and the collected samples were immediately used for analysis. The data for each group were obtained from 3 individual plants. Data of three independent experiments were used to analyze the SA and JA content.

## Supplementary Information

**Additional file 1: Table S1.** Identification and Analysis of Physical and Chemical Properties of ERF

**Additional file 2: Figure S1.** The target fragment for *SlERF2* gene silencing was designed by the SGN VIGS tool.

**Additional file 3: Table S2.** Primers used in the text

## Data Availability

The protein sequences of tomato *ERF* transcription factor family genes were downloaded from PlantTFDB (http://planttfdb.cbi.pku.edu.cn). All sequences were identified by using the online protein structure prediction tool SMART (http://smart.embl-heidelberg.de/). The datasets supporting the conclusions of this article are included with in the article and its Supplementary files.
