# Supplementary figures and images for: Genome-wide identification and functional analysis of the ERF2 gene family in response to disease resistance against Stemphylium lycopersici in tomato
Source: BMC Plant Biol. 2021 Feb 2;21:72. doi: 10.1186/s12870-021-02848-3 (PMC7856819; doi:10.1186/s12870-021-02848-3)

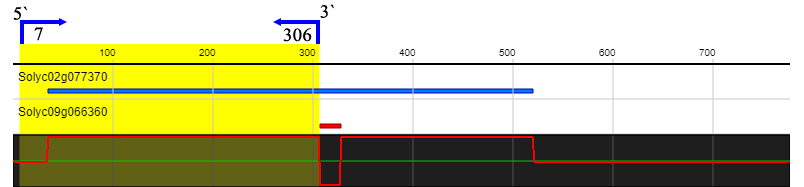

Supplement: Supplementary file 2 — Additional file 2: Figure S1. The target fragment for SlERF2 gene silencing was designed by the SGN VIGS tool. [file 12870_2021_2848_MOESM2_ESM.jpg]
